# Devolved jurisdictions: experience from early research users of family justice data in SAIL Databank.

**DOI:** 10.23889/ijpds.v7i3.1881

**Published:** 2022-08-25

**Authors:** Ludivine Garside, Helen Hodges

**Affiliations:** 1 University of Bristol

## Objectives

This session will share user experience with hosted datasets across two jurisdictions. Family justice is a new domain on the SAIL Databank. Given the devolved policy agenda, challenges arose at different stages in the process of deriving an analytical dataset to compare how children leave care in England and Wales.

## Approach

Details of applications to family courts, and their legal outcomes, were first provisioned in SAIL in 2020 by the respective Children and Families Court Advisory and Support Services (Cafcass) in England and Wales. The analytical challenge was the structure of the datasets, which are fully relational and require information to be summarised and restructured for analysis at case-, application- or child-level. The added complexity of cross-jurisdictional datasets will be examined along four dimensions at planning stage, set-up and life course of the project: data owners’ approvals, data provisioning, metadata, and data linkage within the host platform.

## Results

Early dialogue reduced project risk by aligning timelines with datasets availability and project restrictions, including ethical and GDPR constraints. Metadata allowed the availability and quality of variables to be gauged, alongside the data linkage necessary for analysis. Once data access started, assessing data consistency in each database was the most time-intensive element. High quality metadata can shorten this, and bypass data quality queries. Further in the project life course, clear responsibilities for metadata and data quality ensured that user queries were better targeted. Loss from linkage was partially compensated by the fact that children unsuitably linked remained visible, so researchers could report on which types of families and cases were unmatched, and beyond the project inform about matching rates to be expected from similar linkages.

## Conclusion and Relevance

An important feature of the Cafcass provision in SAIL is the final approval by data owners for researchers to access and link to other datasets in SAIL. With further datasets expected, new linkage opportunities offer excellent prospects for refining comparisons of family justice across England and Wales.

